# A Canard

**Published:** 1884-07

**Authors:** 


A CANARD.–The report that the anonymous novel, *The Bread-Winners*, was offered as a model for a revised materia medica to the Institute is not true. Neither Dr. Hughes nor Dr. Dake wrote it. Their novel will appear later and be just as visionary.

